# Genomic Characterization of Nipah Virus, West Bengal, India

**DOI:** 10.3201/eid1705.100968

**Published:** 2011-05

**Authors:** Vidya A. Arankalle, Bhaswati T. Bandyopadhyay, Ashwini Y. Ramdasi, Ramesh Jadi, Dilip R. Patil, Mehebubar Rahman, Monalisa Majumdar, Parthasarthi S. Banerjee, Amiyakumar K. Hati, Ramaprasad P. Goswami, Dhruba Kumar Neogi, Akhilesh C. Mishra

**Affiliations:** Author affiliations: National Institute of Virology, Pune, India (V.A. Arankalle, A.Y. Ramdasi, R. Jadi, D.R. Patil, A.C. Mishra);; Calcutta School of Tropical Medicine, Kolkata, India (B.T. Bandyopadhyay, M. Rahman, M. Majumdar, P.S. Banerjee, R.P. Goswami, D.K. Neogi);; Gautam Laboratories, Kolkata (A.K. Hati)

**Keywords:** Nipah virus, viruses, full genome sequence, India, intrafamilial spread, zoonoses, dispatch

## Abstract

An intrafamilial outbreak in West Bengal, India, involving 5 deaths and person-to-person transmission was attributed to Nipah virus. Full-genome sequence of Nipah virus (18,252 nt) amplified from lung tissue showed 99.2% nt and 99.8% aa identity with the Bangladesh-2004 isolate, suggesting a common source of the virus.

Nipah virus (NiV) causes encephalitis or respiratory signs and symptoms in humans, with high death rates (*1**–**4*). NiV outbreaks have been reported from Malaysia, Bangladesh, Singapore, and India (*1**,**5**–**13*). Cases in humans have been attributed to zoonotic transmission from pigs and bats (*1**,**14*). We describe a full genome sequence of NiV from an outbreak in India.

## The Study

During April 9–28, 2007, five persons became ill and died within a few days at village Belechuapara, Nadia district, West Bengal, India, which borders Bangladesh. The index case-patient (case-patient 1) was a 35-year-old male farmer addicted to country liquor derived from palm juice. Hundreds of bats were observed hanging from the trees around his residence, which strongly suggested association with the infection of the index case-patient and possibility of contamination of the liquor with bat excreta or secretions.

Three patients were close relatives of case-patient 1: his 25-year-old brother (case-patient 2), his 30-year-old wife (case-patient 3), and his 39-year-old brother-in-law (case-patient 4). They became ill 12, 14, and 14 days, respectively, after contact with case-patient 1. In another person, a 28-year-old man (case-patient 5) who collected blood samples from and performed a computed tomography scan of the brain of case-patient 1, symptoms developed 12 days after contact.

No samples were available from the first 2 case-patients. Brain and lung tissues from case-patient 3, cerebrospinal fluid (CSF) from case-patient 4, and urine and CSF from case-patient 5 were collected. Blood samples were obtained from case-patients 4 and 5 and from 34 asymptomatic contacts from the village. Serum samples from these persons were tested for immunoglobulin (Ig) M and IgG antibodies to NiV (IgM/IgG anti-NiV) with ELISA by using reagents provided by the Centers for Disease Control and Prevention (Atlanta, GA, USA). To detect NiV RNA, urine (250 µL), CSF (100 μL), or autopsied brain or lung tissue (100 mg) were used, and RNA was extracted by using TRIzol LS and TRIzol reagents (Invitrogen Life Technologies, Carlsbad, CA, USA). Nested reverse transcription–PCR (RT-PCR) was conducted by using nucleocapsid (N) gene–based primers (*8*).

Attempts to isolate NiV in Vero E6 cell lines or infant mice were unsuccessful. The full-length genomic sequence was obtained from the lung of case-patient 3 by using 36 sets of primers (Table A1), Superscript II RNase reverse transcriptase for reverse transcription (Invitrogen), and Pfx polymerase for amplification (Invitrogen). The PCR products of predicted molecular size were gel eluted (QIAquick PCR Purification Kit; QIAGEN, Hilden, Germany) and sequenced by using BigDye Terminator cycle sequencing Ready Reaction Kit (Applied Biosystems, Foster City, CA, USA) and an automatic Sequencer (ABI Prism 3100 Genetic Analyzer; Applied Biosystems). PCR products were sequenced in both directions. To determine the genotypic status, phylogenetic analysis was conducted by using partial N gene and full NiV genome sequences with the Kimura 2-parameter distance model and neighbor-joining method available in MEGA version 3.1 software (www.megasoftware.net). The reliability of phylogenetic groupings was evaluated by the bootstrap test with 1,000 bootstrap replications.

Patients’ signs and symptoms included high fever (103°F–105°F [39.4°C–49.6°C]) with and without chills, severe occipital headache, nausea, vomiting, respiratory distress, pain in calf muscles, slurred speech, twitching of facial muscles, altered sensorium, (focal) convulsions, unconsciousness, coma, and death. The first 3 case-patients died within 2–3 days after symptom onset; case-patients 4 and 5 died after 5 and 6 days, respectively. Clinical investigations could be conducted for case-patients 4 and 5. Results of serologic tests for malaria parasite, typhoid, anti-dengue IgM, HIV, and hepatitis B surface antigen were negative. Peripheral blood profiles were within reference limits. Alanine aminotransferase, aspartate aminotransferase, creatine phosphokinase, and C-reactive protein were elevated. Blood gas analysis in case-patient 4 (case-patient 5 values in parentheses) showed oxygen saturation 49.4% (71%), pO_2_ 36.1 mm Hg (44.8 mm Hg), pCO_2_ 44.4mm Hg (44.1 mm Hg), HCO_3_ 15.7 mmol/L (19.1 mmol/L), and pH 7.166 (7.255). Lumbar puncture of case-patient 5 showed opening pressure within reference range (1 drop/second), 2 cells/cm; all cells observed were lymphocytes. Chest radiograph indicated pulmonary edema, which suggested acute respiratory distress syndrome. At the time of admission, computed tomography and magnetic resonance imaging scans of the brain showed no abnormality.

Serum samples from case-patients 4 and 5 were positive for IgM anti-NiV. Of the clinical samples screened, brain and lung tissues of case-patient 3, CSF of case-patient 4, and urine of case-patient 5 were NiV RNA positive. Of the 34 asymptomatic contacts, 1 was positive for IgG anti-NiV and negative for IgM anti-NiV and did not report any major illness in the past. This positivity may reflect a previous subclinical infection or cross-reactivity in ELISA needing further follow-up.

Before this report, similar cases had not been reported from the village or the surrounding area. Partial N gene sequences confirmed NiV in the clinical specimens from all 3 case-patients. Phylogenetic analysis showed that similar to findings from the 2001 outbreak study (*8*), viruses from Bangladesh and India clustered and diverged from the viruses from Malaysia. (Figure, panel A). The length of the full genome of the isolate from India was 18,252 nt. The sequence of this virus (INDNipah-07–1, GenBank accession no. FJ513078) was closer to the virus from Bangladesh (Figure, panel B), with 99.2% (151 nt substitutions) and 99.80% (17 aa substitutions) identity at nucleotide and amino acid levels respectively. Of the 151 nt substitutions, 9 occurred in the N open reading frame (ORF),11 in the phosphoprotein ORF, 8 in the matrix ORF, 11 in the fusion glycoprotein ORF, 7 in the attachment protein ORF, and 47 in the large polymerase ORF. Fifty-eight substitutions occurred in nontranslated regions at the beginning and the end of each ORF. The intergenic sequences between gene boundaries were highly conserved in the isolate from India, compared with the isolate from Bangladesh, which showed 1 change (GAA to UAA) between the attachment protein and large polymerase genes. No change was observed in the leader and the trailer sequences.

**Figure:**
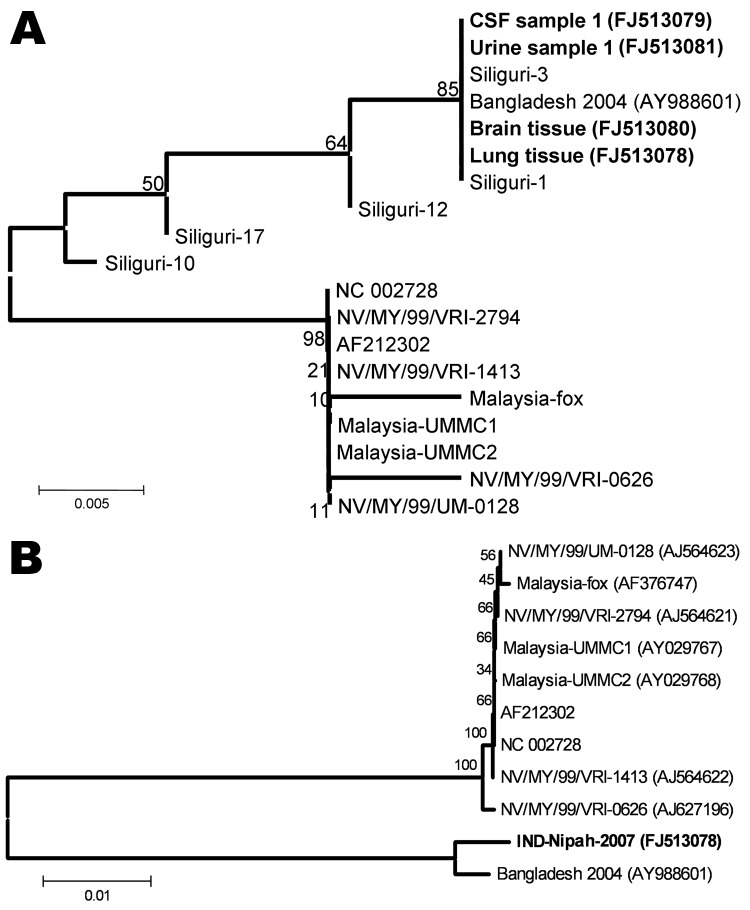
A) Phylogenetic analysis based on partial nucleocapsid (N) gene nucleotide sequences (159 nt, according to Nipah virus [NiV] Bangladesh sequence, GenBank accession no. AY988601, 168–327 nt) of the 4 NiVs sequenced during this study (**boldface**). Five sequences of the viruses from Siliguri (*8*) and from representative NiV sequences obtained from GenBank indicated by the respective accession numbers. Values at different nodes denote bootstrap support. B) Full genome–based phylogenetic analysis of the NiV sequenced from the lung tissue of a patient (**boldface**). Representative NiV sequences obtained from GenBank are indicated by the respective accession numbers. Values at different nodes denote bootstrap support. Scale bars indicate nucleotide substitutions per site.

The Table compares amino acid substitutions in the different regions of the genome of the isolate from India with those of the viruses from Bangladesh and Malaysia. Of the 17 aa substitutions, 7 were unique to the isolate from India, and 10 were similar to the isolates from Malaysia. Overall, however, the isolate from India was closer to the isolate from Bangladesh, although distinct differences were observed.

**Table:** Regionwise amino acid substitutions in the Nipah virus genome*

Region and amino acid position	India	Bangladesh	Malaysia
Phosphoprotein			
228	**K**	R	R
276	**S**	G	G
285	R	H	R
310	**R**	G	G
Nucleocapsid protein			
188	E	D	E
211	**R**	Q	Q
Matrix protein			
13	**I**	M	M
Fusion protein			
19	**I**	M	M
207	L	S	L
252	D	G	D
Attachment protein			
304	**V**	I	I
Large polymerase protein			
94	I	T	I
112	K	R	K
632	N	S	N
639	N	D	N
665	T	I	T
1748	I	V	I

*GenBank accession numbers of isolates examined: India (FJ513078), Bangladesh (AY988601), and Malaysia (AY029767,AY029768, and AJ564623). **Boldface** indicates unique amino acids in the isolate from India.

To our knowledge, this is the second report of an NiV outbreak in India, identified within 1 week of the investigation. The first outbreak affected mainly hospital staff or persons visiting hospitalized patients; the 74% case-fatality rate strongly suggested person-to-person transmission (*8*). Both outbreaks (2001 and 2007) occurred in the state of West Bengal bordering Bangladesh wherein several outbreaks of the disease have been reported (*7**,**9**–**13*). However, fruit bats from West Bengal have not been screened for evidence of NiV infection. This state needs to create awareness about NiV and obligatory testing of suspected case-patients.

## Conclusions

NiV caused an intrafamilial outbreak with a 100% case-fatality rate, which confirmed person-to-person transmission. The NiV strains from India and Bangladesh were closer than the Malaysian viruses. Although the outbreaks occurred in neighboring geographic areas, NiV outbreaks in Bangladesh and India were not caused by the same virus strain or by spillover.

## Appendix

**Table A1:** Primers used for full genome amplification and sequencing of Nipah virus in an interfamilial outbreak, West Bengal, India, 2007

Region	Forward primer, 5′ → 3′	Reverse primer, 5′ → 3′
Leader sequence and nucleocapsid protein	F1 1–ACCAAACAAGGGAAAATATGGAT-23	R1 287-CTCGGCTGCACTCGGAGATCTGAT-310
	F2 17–TATG GATACGTTAA AATATAT-37	R2 590-TGATAACTGCAGAGACCAGAGT-611
	F3 507–TCAA GACTGCTCGG GACAGCAG-528	R3 1115-TCAATAGTAGTAGCCACACCCAT-1137
	F4 999–GC TTGATGCTAC TCTACAGAG-1019	R4 1595-TGATTGCTGGGTCATCTGTTGCATT-1619
	F5 1479-T GACAGTGTGC CGAGCAGTTCTGT-1503	R5 2115-TAAGAGGTATTGTATACTCCAGT-2137
	F6 1999-CA CACTACACTC TAATAAAGAT-2020	
Phosphoprotein	F7 2551-TGCAGTGCAC CAGTGGAGAA TCT-2573	R6 2594-CACCTCCATCATCCTTAGAC-2613
	F8 3017-TTCT CCTGTGATTG CTGAACACT-3039	R7 3081-TCCAAATTGACATTTCTGTTAGT-3103
	F9 3528-GAT AAGACTGAGATCACCAGCGA T-3551	R8 3627-CAGATGGATATTTCTCGTCTGT-3648
	F10 4054-CTTGAGTC TATTGACAGG GTTCT-4075	R9 4176-TCACTGGTTTAAGCTCAGGATT-4197
	F11 4576-TCTTC TACATGTAGA CAGTAG-4596	R10 4692-TCATGCTACATACACTTCAAT-4712
Matrix protein	F12 5125-GAGCAT TTCAAGTGAG TCT-5143	R11 5181-TTATCAAGATACCCACCATTCT-5202
	F13 5586-TTGAT CAGATACAGC TCGAC-5605	R12 5662-TAGTTCGTGGAATCATGTAGATT-5684
	F14 6091-GCCTTCTGTT CCGAGAGAGT T-6111	R13 6123-TGTATTGTCAATGAAGACATCAT-6145
Fusion protein	F15 6621-GTTGGTAGAC CTATCAATCA TAT-6643	R14 6713-CACTGCACTCCGAGATCATC-6732
	F16 7105-GCAGCA TAGAATCAAC TAATG-7125	R15 7161-GCGGTCAGTACATAGACTGT-7180
	F17 7522-TCCAACAGG CCTATATCCA AG-7542	R16 7570-TGCTGATCCATTCTGAATTGT-7590
	F18 8078-GCT CAACGACTCCTTGATACTGT T-8110	R17 8130-TACAGTATGATCATAGACAACAT-8152
	F19 8638-TGTACTTGCAATT ATACATTGT-8659	
Attachment protein	F20 9121-GATCCATTGT AATCATAGTG-9140	R18 8723-TATCCAATGAGTTATGGACCT-8743
	F21 9630-T ACTTTGCATA TAGCCACCTG-9650	R19 9202-TGCTGGATACTCTGCAATGCAT-9223
	F22 10099-TC GCAGAGTGTC AATACAGCAA ACCT-10124	R20 9698-TGTCTAGTACCTCTCCAACTCCT-9720
	F23 10531-GCAACCAGAC CGCAGAGAAT CCT-10553	R21 10149-TATAATGACTGTTTGGTCTAAT-10170
	F24 11062-TCAGAGTTA ACAGTCTATA CAT-11083	R22 10561-TACTTCATTATCTTTGAATACAG-10583
		R23 11104-CATTGATTGTCATCACTATGC-11124
Large polymerase protein	F25 11668-TGC ATATTGCGTA CCCTGAATGT-11690	R24 11724-TGTTATCAAGTTTGCTAGTCAT-11745
	F26 12151-GGATGATGAT GGAGACAACA AT-12172	R25 12200-GAGGGCATTGGACCTCGAGATT-12221
	F27 12681-GCACATGCAT CTAAGCATAT-12700	R26 12738-TTCCCAGTTCTTGACACAATCATC-12761
	F28 13221-TCAGTTCCTC GTGGAAACAG TC-13241	R27 13252-TATATTATTGATGGATTGAGGAT-13274
	F29 13776-GATGA TATATTCATT CATTATCCT-13779	R28 13859-TCTCATAGGCACTCAAGAAT13878
	F30 14244-TCAAGGA ATGTCGGCTA TTGTAT-14266	R29 14287-TCAGTTGATATAAGGAGTTGCT-14308
	F31 14728-TAG CTAGCTTCCT GATGGACAGG-14750	R30 14770-TTGTCCAGTATCTCATGAGCGG-14791
	F32 15241-GTACAGATGA GAGATCAGAT AT-15262	R31 15304-TACTGTCGCAATCCTGATAGCAG-15326
	F33 15791-TGATCCAGAT CCTGTTTCAG-15810	R32 15872-TGAAGCTCCTCAGTTGACCAT-15892
	F34 16381-CTGTGATTAA CCTACGAGAG GATAT-16405	R33 16461-TTCAGATCTATTATCCAAGGAGG-16483
	F35 17038-CAGGTCAGAGAGA ACTGAAGCT-17059	R34 17064-TCAGCAATCGAGTATTCGGATGG-17086
	F36 17371-TCTCAAGATT ATTTAACATG T-17391	R35 17429-TAGAATCTGGGTTGCTATACACT-17451
	F37 17831-TTCACATCAT TTGGAACCGT AT-17852	
Trailer sequence		R36 18230-ACCGAACAAGGGTAAAGAAGAAT-18252
